# Lateral lumbar and thoracic interbody fusion (LLIF) for thoracolumbar spine trauma (Trauma LLIF): A single-center, retrospective observational cohort study

**DOI:** 10.1016/j.xnsj.2024.100534

**Published:** 2024-07-27

**Authors:** Daniele Gianoli, Linda Bättig, Lorenzo Bertulli, Thomas Forster, Benjamin Martens, Martin N. Stienen

**Affiliations:** a Spine Center of Eastern Switzerland, Kantonsspital St. Gallen & St. Gallen Medical School, St. Gallen, Switzerland; b Department of Orthopedic Surgery, Kantonsspital St. Gallen & St. Gallen Medical School, St. Gallen, Switzerland; c Department of Neurosurgery, Kantonsspital St. Gallen & St. Gallen Medical School, St. Gallen, Switzerland

**Keywords:** Lateral lumbar interbody fusion, Lateral thoracic interbody fusion, Thoracolumbar fractures, Kyphosis, Complications, Outcome, XLIF

## Abstract

**Background:**

Pain, disability and progressive kyphosis is a common problem after traumatic injury of the thoracolumbar (TL-) junction. Surgical treatment may include long-segment posterior or short-segment anterior-posterior fusion. We aim to report our experience with the application of short-segment posterior instrumented fusion with anterior column support using lateral lumbar or thoracic interbody (LLIF) cages.

**Methods:**

In this retrospective, single-center observational cohort study we included consecutive patients treated surgically for traumatic injury of the TL-junction (Th10/11-L2/3) by posterior instrumentation/fusion and LLIF. We measured segmental kyphosis, complications, and outcomes until last follow-up (about 3 years postoperative).

**Results:**

We identified 61 patients (mean age 39.0 years [SD 13.3]; 23 females [37.7%]) with A3 fractures without (n=48; 78.7%) or with additional sagittal split component n=11; 18.0%. Additional posterior tension band injury was present in n=26 (42.6%). The affected levels of injury were Th12/L1 in n=25 (41.0%) and Th11/12 in n=22 (36.1%). The segmental kyphotic angle was 14.6° (6.7°) preoperative and remained significantly reduced at all times of follow-up at discharge (5.4°±5.5°; p<.001), at 90 days (7.2°±5.5°; p<.001), after partial hardware removal (7.2°±6.0°; p<.001) and at last follow-up (8.1°±6.3°; p<.001). We noticed a tendency for less progression of kyphosis in the group with 2-staged, compared to single-staged bisegmental surgery (mean difference (MD) 3.1° after partial hardware removal, p=.064). During follow-up, n=11 experienced complications (18%), n=58 (95.1%) had an excellent or good outcome and solid fusion was noticed in n=60 (98.4%).

**Conclusions:**

“Trauma LLIF” should be considered as possibility for short-segment anterior-posterior fusion for injuries of the TL- junction. We observed most reproducible and long-lasting kyphosis reduction with a temporary bisegmental, 2-staged procedure resulting in monosegmental fusion (posterior instrumentation/fusion with delayed LLIF and partial hardware removal to release the noninjured caudal motion segment).

## Introduction

The thoracolumbar (TL-) junction is a mechanical transition between the rigid thoracic and the flexible lumbar spine. It is the most common site of fractures to the spine [1,2]. About 60%–70% of TL-injuries involve regions between the 10th thoracic (T10) and 3rd lumbar (L3) vertebra [3]. The types of TL-injuries mechanisms include compression (A-type injuries), flexion-extension (B-type injuries) and translation/rotation (C-type injuries) [3,4]. In absence of a neurological deficit, most A-type and some of the B-type injuries may be treated conservatively [5]. However, if the vertebral body trauma is extensive, a neurological deficit is present, clear disruption of the posterior ligamentous complex is evident or segmental kyphosis is more than 15°, the injury can be considered unstable and surgical treatment is often recommended. The ideal surgical management of TL-fractures is still discussed, including the best strategy to promote fusion (posterior only vs. anterior only vs. combined anterior-posterior) [6], the approach (traditional open vs. Wiltse vs. percutaneous approach), and the extent of fixation (short-segment vs. long-segment fixation, fusion vs. instrumentation) [3,[7], [8], [9], [10], [11], [12], [13], [14], [15]].

Today, posterior short-segment internal fixation with pedicle screws (open or percutaneous) is a common approach to surgically address a TL-fracture [13,16]. This technique involves pedicle screw fixation 1 level above and 1 level below the fracture. Due to the injury of the intervertebral disc (IVD) and lack of anterior support in posterior only procedures, there is a considerable rate of hardware failure and postoperative kyphosis with bisegmental instrumentation/fusion [17,18]. In the last years, minimally invasive techniques have been developed to allow for relatively atraumatic replacement of the injured IVD through a mini-open lateral approach ((extreme) lateral lumbar or thoracic interbody fusion = XLIF/LLIF) [6,19,20]. Applying such an approach in the trauma setting may help to better maintain the biomechanical integrity and prevent posttraumatic malalignment, progressive kyphosis, neurological function and backpain [21]. For degenerative and deformity indications, the LLIF technique is known for its high fusion rate, low chance of cage subsidence and powerful abilities to correct segmental deformities including scoliosis and kyphosis, which may allow to treat some of these injuries with monosegmental fusion [20,22,23]. The potential of LLIF, when applied for unstable TL-injuries, has not been studied and reported in the scientific literature to date.

The aim of this paper is to describe our experience with LLIF for monosegmental fusion in the setting of TL-trauma over the past years, and to compare the radiological and clinical outcomes of different surgical strategies.

## Material and methods

### Hospital setting

The Kantonsspital St.Gallen is an academic, tertiary teaching-hospital, associated with the Medical School of St.Gallen, Switzerland. It serves a population of approximately 1′000’000 inhabitants. The Spine Center of Eastern Switzerland is formed mutually by 12 board-certified Neurosurgeons or Orthopedic spine surgeons and 7 residents/physician assistants. It is certified as Surgical Spine Center of Excellence by the Eurospine and as AO Spine center. Use and types of implants are unified. About 1100-1300 spine-surgical procedures under general anesthesia are performed annually. LLIF with static cages was introduced at our center in 11/2011 and is performed about 50-60 times per year on average, mostly for degenerative disc disease and deformity indication. LLIF procedures in this series were performed by 9 senior spine surgeons, or by fellows or senior residents under supervision.

### Ethical considerations

The institutional review board (IRB) of St.Gallen approved the study (BASEC ID 2023-01343). Retrospective collection, analysis and publication of anonymized patient data was allowed with an institutional waiver for informed consent.

### Patient identification, in- and exclusion criteria

**Fig. 1:**
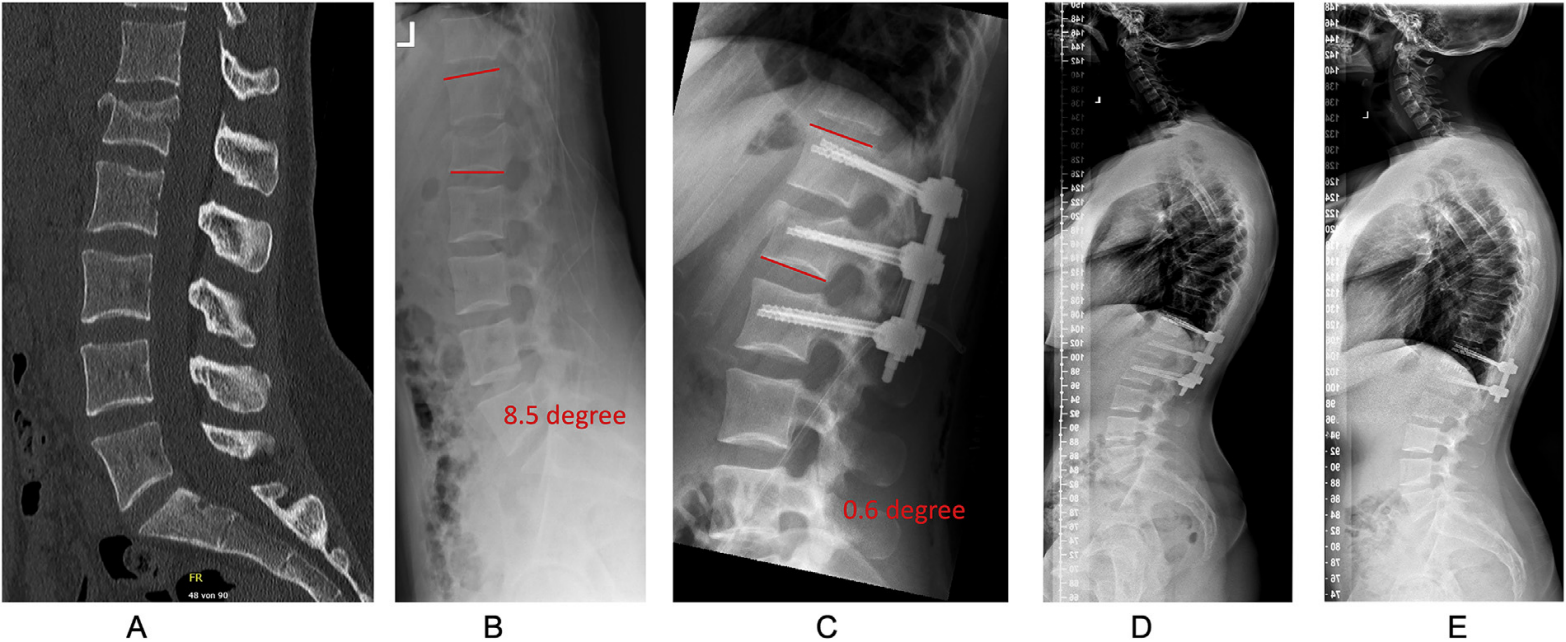
Case vignette of a typical patient treated with 2-staged, temporary bisegmental “Trauma LLIF”. This 26-year-old female suffered from a fall off a horse with impact on the spine. (A) Sagittal computed tomography revealed an incomplete burst fracture of L1 (AO Spine type A3) without evident injury of the posterior tension bands. (B) Standing lateral x-ray studies show a kyphosis of 14 degree at the vertebral body of L1 and of 8.5 degree as segmental kyphosis angle over the motion segment Th12/L1 (illustrated in red). (C) Standing lateral x-ray studies of the thoracolumbar junction 2 days after posterior instrumented fusion of Th12/L1 with temporary instrumentation of L2 show a reduction of the segmental kyphosis to 0.6 degree (illustrated in red). (D) Whole-spine x-ray studies at 90 days. The clinical and radiological course is uneventful; hence the patient is scheduled for additional “Trauma LLIF” and release of the motion segment L1/2 shortly after. (E) Whole-spine x-ray studies at 18 months after the posterior surgery and 14 months after the “Trauma LLIF” of Th12/L1 and removal of the temporary instrumentation to L2. The patient reported no significant pain or disability. She was working 100% without limitations.

**Fig. 2:**
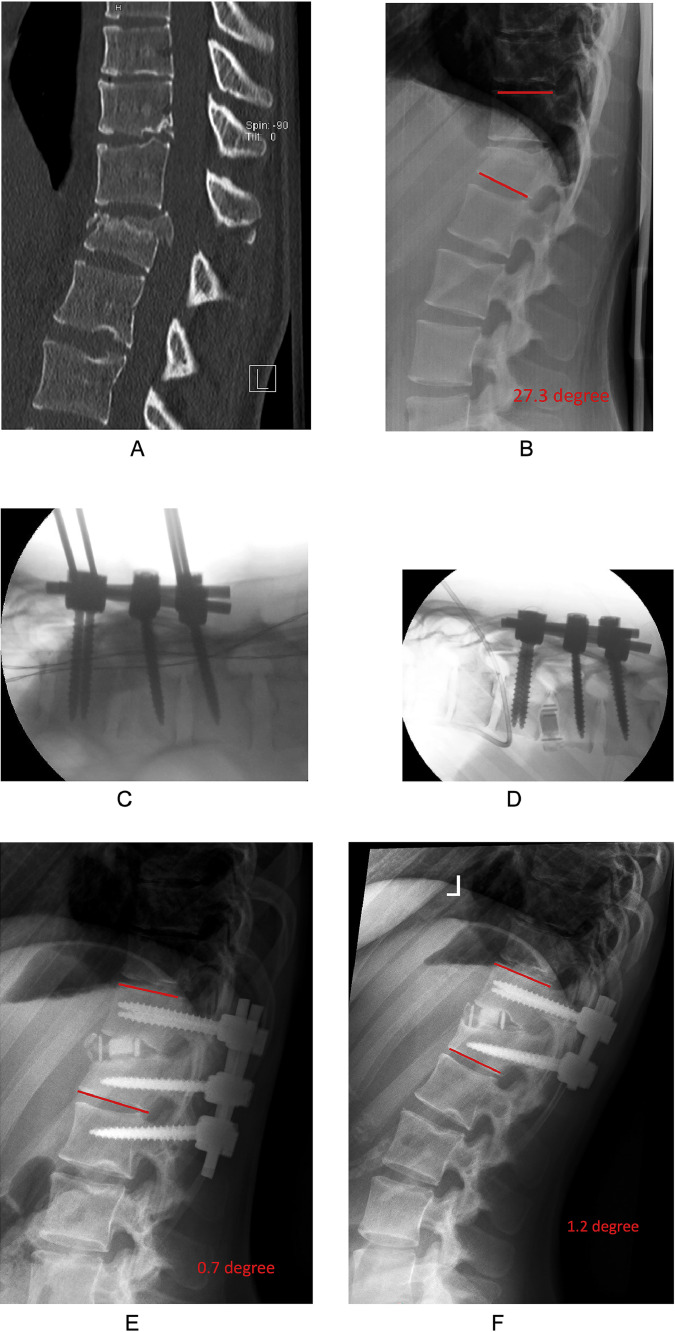
Case vignette of a typical patient treated with single-staged, temporary bisegmental “Trauma LLIF”. This 19-year-old female was hit by a car with impact on the ground. (A) Sagittal computed tomography revealed an incomplete burst fracture of L1 (AO Spine type A3) with signs of injury of the posterior tension bands. (B) Sitting x-ray studies of the thoracolumbar region demonstrate the enlarged interspinous distance and a segmental kyphosis Th12/L1 of 27.3 degree (illustrated in red). (C) The surgical treatment included an open posterior fusion of Th12/L1 with iliac graft and instrumentation of L2. (D) During the same anesthesia, the patient was positioned in lateral decubitus position and a 45mm long and 10mm high interbody cage with 10 degree of lordosis was inserted, to compensate for the kyphosing endplate fracture of L1. (E) Standing x-ray studies before discharge reveal a reduction of the segmental kyphosis Th12/L1 to 0.7 degree (illustrated in red). The patient recovered well and was reoperated 4.5 months after the initial surgery with shortening of the rods and removal of the temporary pedicle screws of L2. (F) At the final follow-up at our institution about 1.5 years postoperative, the patient was doing well without obvious complications (1.2 degree segmental kyphosis at Th12/L1 – illustrated in red).

It was our aim to identify patients undergoing single-level “Trauma LLIF” for attempted monosegmental fusion of a vertebral body fracture with or without B- or C-type injury of the TL-junction (Th10/11-L2/3; Figs. 1 and 2). We searched our electronic operation program to identify 87 patients between May 2011 and November 2023, who received an LLIF procedure in the setting of a traumatic injury of the spine. Of those, 26 patients were excluded for the following reasons: Trauma LLIF was performed at 2, 3 or more levels (n=9), Trauma LLIF was performed in a delayed fashion, after failed conservative therapy (n=7), individualized treatment (not based on our usual Trauma LLIF standard), e.g., because of prior surgeries, ankylosing conditions of the spine or other, significant comorbidities (n=6), Trauma LLIF was performed at a different segment than Th10/11-L2/L3 (n=1), loss of follow-up with patient living abroad (n=2) or insufficient baseline imaging available (n=1).

### Indication and surgical treatment

Most neurologically intact patients seen with traumatic injuries in the TL-junction and intact tension bands were successfully treated conservatively. Indications for surgical treatment with Trauma-LLIF included confirmed B- or C-type injury, or A-type injury in patients with significant segmental kyphosis (>15°), as well as patients with relevant injury-related compromise of the spinal canal (retropulsed posterior wall osseous fragment or traumatic disc herniation)—especially in case of neurological signs or deficits. Moreover, patients with severe mechanical pain, which does not allow for mobilization within 2–3 days despite adequate analgesia, were also offered surgery.

For the Trauma-LLIF technique, we considered patients with A1, A2 and A3 fractures, according to the AO Spine classification. Patients with a superior burst-split fracture (A3.2.1 according to Magerl [3]; A4 according to the AO Spine classification) [4] were also considered in case of low fracture comminution, where a single simple split fracture line of the fractured vertebral body (without disc material ruptured into the vertebral body) was evident down to the lower disc level.

In this series the surgical strategy was not standardized *à priori* but was chosen individually for each case, considering factors such as patient age and bone quality, fracture type and morphology, comorbidities and concomitant injuries, working status of the patient, and skills of the surgeon on call. Over the 12 years of experience with Trauma LLIF we have developed a more standardized approach with “2-staged, temporary bisegmental Trauma LLIF,” containing a posterior open or percutaneous pedicle-screw based instrumentation/fusion of the injured motion segment, with angle-stable distraction and temporary additional instrumentation 1 level below (Fig. 1). Patients are followed at 6 and 12 weeks (90 days), and after partial healing of the endplate defect at this time, the Trauma LLIF procedure is scheduled in a delayed fashion around 3–5 months after the trauma to promote solid interbody fusion (Fig. 1). The temporary additional instrumentation of the lower level is removed in the same session by minimal opening of the posterior incision and bilateral cutting of the rod (Figs. 1 and 2). A CT scan of the surgical site is conducted at 12 weeks and prior to scheduled Trauma LLIF/partial hardware removal to confirm the posterior fusion is taking place and no hardware loosening is evident. For Th12/L1 or higher, the procedure is usually done trans-pleural and requires placement of a thoracic drain for 24–48 h. No neuromonitoring is used for the lateral procedure in the upper lumbar spine (L1-L3).

In some patients, we combined a primary posterior-anterior Trauma LLIF procedure during the index hospitalization with temporary additional instrumentation of the lower level (=single-staged, temporary bisegmental; Fig. 2). Those patients were usually re-scheduled for release of the lower motion segment around 3–6 months after the index surgery, or later.

**Fig. 3:**
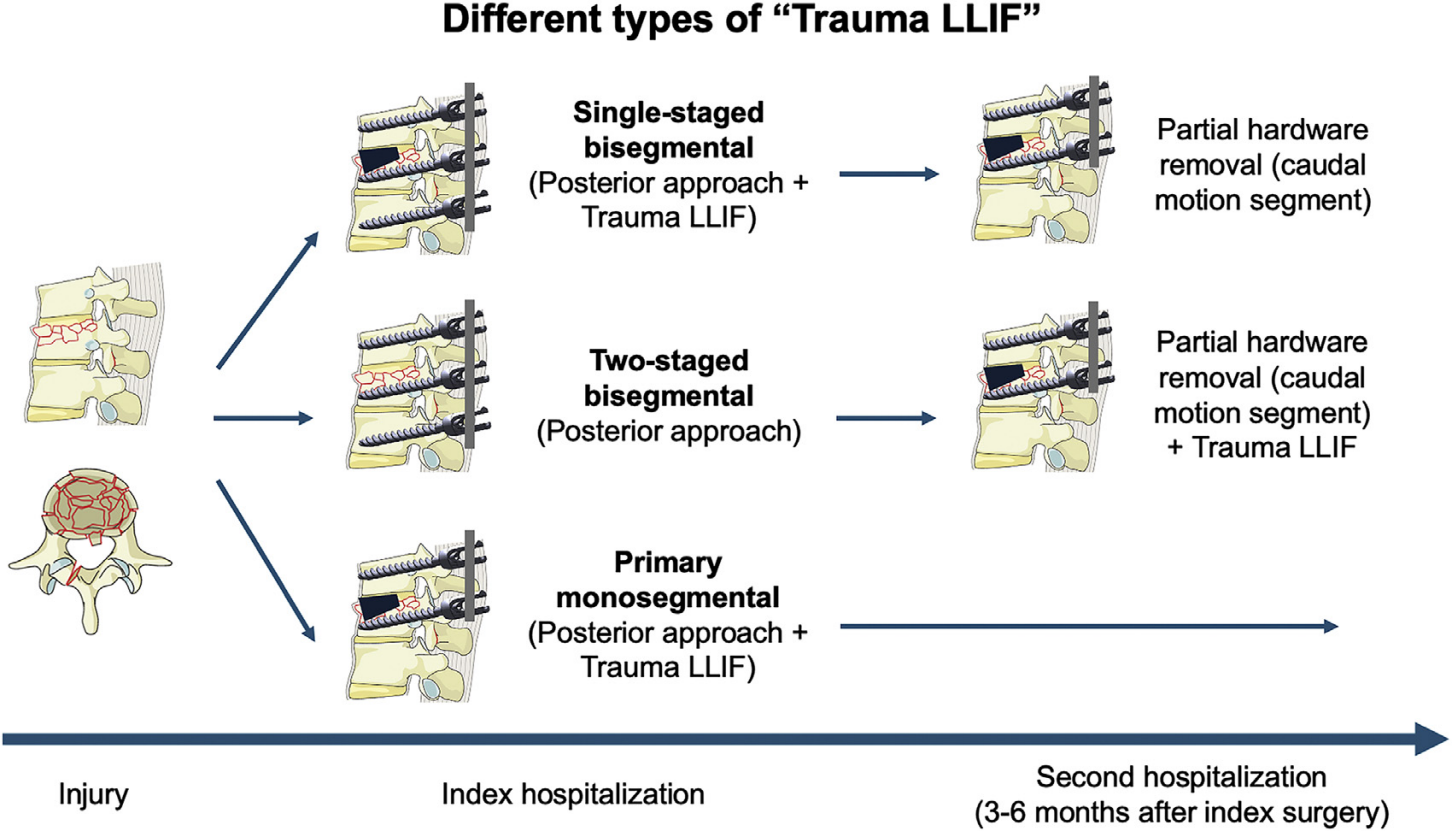
Scheme and timeline explaining the 3 different types of “Trauma LLIF” – single-staged bisegmental, 2-staged bisegmental and primary monosegmental. Note that patients undergoing bisegmental instrumentation were seen back in clinics around 90 days with a CT-scan to plan the partial hardware removal (with Trauma LLIF in those with 2-staged surgery) between 3 and 6 months after the first operation. The schematic illustration of the incomplete burst fracture is adopted from the AO Spine.

In some patients with suspected sufficiently high bone quality, single-staged, primary monosegmental procedures without temporary instrumentation of the next lower motion segment were considered (posterior-anterior) during the index surgery to avoid a 2nd hospitalization and surgery for the LLIF & hardware removal. All 3 strategies are illustrated on a timeline in Fig 3.

### Variables and statistical considerations

Descriptive statistics were used to characterize the sample and surgery-specific aspects. Our main dependent variable of interest was the segmental kyphosis angle (in °), determined as the sagittal Cobb angle between the upper endplate of the upper vertebra and the lower endplate of the lower vertebra of the injured segment (see Figs. 1B and 2B). Segmental kyphosis was measured preoperative, as well as at all time points during follow-up. Pseudarthrosis was defined as lack of confirmed segmental fusion or bone ingrowth of the cage (in computed tomography imaging, whenever available), with or without screw loosening or delayed onset of back pain that led to a reoperation. Moreover, we determined complications between surgery and 90 days postoperative, as well as between 90 days and last follow-up in all patients. The severity of complications was determined by the Therapy-Disability-Neurology (TDN) grading scale [24]. As Patient-Reported Outcome Measures (PROMs) were only introduced at our center in 2022, we used the MacNab criteria (excellent; good; fair; poor) to estimate the clinical/functional outcome at 90 days and last follow-up.^25^

Paired t-tests were used to compare segmental kyphosis at each time point of follow-up, compared with preoperative. Our independent variables of interest were single- vs. 2-staged procedures, monosegmental vs. bisegmental procedures, younger vs. higher age (determined by median of the total cohort; 36.0 years), sex, presence of AO Spine B- or C-type injury and higher degree of vertebral destruction (complete burst fracture). T-tests were used to compare segmental kyphosis at each time point between study groups. Logistic regression models were built to estimate the effect size between patient- or disease-specific variables and outcome at 90 days or last follow-up. Results were expressed as odds ratio (OR) with 95% confidence interval (CI).

Stata v18 SE for Mac (StataCorp LLC, College Station, TX [USA]) was used for coding and statistical analysis. P-values of <.05 were considered significant.

## Results

### Sample description

We included n=61 patients with a mean age of 39.0 years (SD 13.3; range 18–71) and the female to male ratio was 23 (37.7%) to 38 (62.3%). Patients were rather healthy, mostly nonsmoking with low American Society of Anesthesiology risk scales, low rates of severe disability (n=55 (90.2%) with Charlson Comorbidity Index of 0 points) and frailty (Table 1). According to the AO Spine TL-trauma classification, 48 patients (78.7%) were operated for A3 fractures; an additional B- or C-type injury was identified in n=24 (39.3%) and n=2 (3.3%), respectively. The mostly injured motion segments were Th12/L1 in n=25 (41.0%) and Th11/12 in n=22 (36.1%; Table 1).

**Table 1:** Demographic baseline information on n=61 patients who underwent lateral lumbar or thoracic interbody fusion (LLIF) for traumatic indications.

Age, in years	39.0 (13.3)
Sex	
Female	23 (37.7%)
Male	38 (62.3%)
Body mass index, in kg/m^2^	24.9 (4.4)
Smoking status	
Smoker	15 (24.6%)
Nonsmoker	33 (54.1%)
Missing data	13 (21.3%)
American Society of Anesthesiology risk scale	
I	33 (54.1%)
II	26 (42.6%)
III	2 (3.3%)
Charlson comorbidity index	
0	55 (90.2%)
1 or higher	6 (9.8%)
Canadian clinical frailty index	
Very fit	33 (54.1%)
Well	23 (37.7%)
Managing well	4 (6.6%)
Vulnerable	1 (1.6%)
Vertebral body injury type*	
A1 – compression fracture	2 (3.3%)
A3 – incomplete burst fracture	48 (78.7%)
A4 – complete burst fracture	11 (18.0%)
Ligamentous injury type*	
B1 – trans-osseous injury	3 (4.9%)
B2 – posterior tension band disruption	21 (34.4%)
C – dislocation or rotational injury	2 (3.3%)
None	35 (57.4%)
Level of injury	
Th10/11	1 (1.6%)
Th11/12	22 (36.1%)
Th12/L1	25 (41.0%)
L1/2	11 (18.0%)
L2/3	2 (3.3%)
**Total**	**n=61 (100%)**

⁎ According to the AO Spine thoracolumbar injury classification. Data is presented as mean (standard deviation) or count (percent).

### Surgical treatment

Table 2 contains information regarding the surgical treatment. For the posterior procedure, percutaneous or minimally invasive transmuscular instrumentation was used in most cases (n=36; 59.0%). In all but 6 cases (9.8%) temporary bisegmental instrumentation/fusion was chosen over initial monosegmental fusion. In n=39 patients (63.9%) the LLIF was conducted during the same hospitalization (after a mean of 2.6 days (SD 3.7)). Depending on the endplate damage, disc height and resulting kyphotic deformity of the fractured vertebra, rigid interbody cages with optimal shape and fit were chosen; usually with 6–10° of lordosis (Table 2). In 6 patients with particular anatomical constellations, hyperlordotic expandable interbody spacers were used. Posterior hardware removal to release and remobilize the adjacent motion segment was performed in n=53/55 patients (96.4%) after a mean interval of 133.9 days (SD 40.7); the remaining 2 patients did not wish to undergo hardware removal.

**Table 2:** Procedure-specific information on n=61 patients who underwent lateral lumbar or thoracic interbody fusion (LLIF) for traumatic indications.

Posterior approach	
Open, conventional	25 (41.0%)
Percutaneous, minimally invasive	36 (59.0%)
Number of motion segments instrumented	
One, e.g., Th11/Th12	6 (9.8%)
Two, e.g., Th11/Th12/L1	54 (88.5%)
Three, e.g., Th10/Th11/Th12/L1	1 (1.6%)
Interval between posterior and lateral procedure*	
Immediate – same hospitalization	39 (63.9%)
Staged – different hospitalization	22 (36.1%)
Treatment type†	
Single-staged, monosegmental	6 (9.8%)
Single-staged, temporary bisegmental	33 (54.1%)
Two-staged, temporary bisegmental	22 (36.1%)
Type of LLIF implant	
Static (NuVasive CoRoent ® or Modulus ®)	52 (85.3%)
Static (J&J Synmesh ®)	2 (3.3%)
Expandable (Globus Medical ELSA ®)	7 (11.5%)
Angle of LLIF implant	
Parallel, 0°	7 (11.5%)
Anatomic, 6–10°	48 (78.7%)
Hyperlordotic, 20–30°	6 (9.8%)
**Total**	**n=61 (100%)**

⁎ The interval between the posterior and lateral procedure was 2.6 days (SD 3.7) in the immediate group and 102.8 (SD 37.8) days in the staged group (p<0.001).

† In 53/55 patients with bisegmental treatment, the noninjured motion segment was released by removal of pedicle screws and shortening of rods after an interval of 133.9 days (SD 40.7) following the initial surgery. Data is presented as mean (standard deviation) or count (percent).

### Segmental sagittal cobb angles and fusion rate

Segmental sagittal angles before and after surgical treatment are illustrated in Table 3. The preoperative segmental kyphotic angle was 14.6° (6.7°) for the total cohort and remained significantly reduced at all times of postoperative follow-up at discharge (5.4° ± 5.5°; p<.001), at 90 days (7.2° ± 5.5°; p<.001), after hardware removal (7.2° ± 6.0°; p<.001) and at last follow-up (8.1° ± 6.3°; p<.001).

**Table 3:** Segmental sagittal angles preoperative and during the postoperative follow-up in n=61 patients who underwent lateral lumbar or thoracic interbody fusion (LLIF) for traumatic indications.

Segmental kyphosis	Preoperative		Discharge*		90-day follow-up†		Follow-up after hardware removal‡		Last follow-up§	
	Angle	p-value	Angle	p-value	Angle	p-value	Angle	p-value	Angle	p-value
Total group	14.6 (6.7)	n/a	5.4 (5.5)	<.001	7.2 (5.5)	<.001	7.2 (6.0)	<.001	8.1 (6.3)	<.001
Bisegmental		.474		.494		.388		.064		.115
single-staged	14.1 (7.0)		5.7 (5.7)		7.7 (5.8)		8.5 (6.2)		9.1 (6.5)	
two-staged	15.4 (6.2)		4.75 (5.5)		6.4 (5.0)		5.4 (5.3)		6.5 (5.7)	
Extend of stabilization		.266		.842	n/a	n/a	n/a	n/a		.413
Bisegmental	14.9 (6.6)		5.3 (5.6)						7.9 (6.1)	
Monosegmental	11.7 (7.4)		5.8 (5.3)						10.2 (7.5)	
Age groups		.611		.424		.221		.204		.151
Age < 36 years	15.0 (5.9)		4.8 (5.1)		6.2 (5.0)		6.1 (5.8)		7.0 (6.0)	
Age > 36 years	14.1 (7.5)		6.0 (5.9)		8.1 (5.9)		8.2 (6.2)		9.4 (6.4)	
Sex		0.520		.004		.021		.049		.056
Female	13.9 (6.9)		2.8 (6.4)		4.8 (6.3)		4.9 (7.0)		6.1 (7.0)	
Male	15.0 (6.6)		6.9 (4.4)		8.4 (4.7)		8.4 (5.1)		9.3 (5.6)	
B- or C-type injury		.086		.938		.938		.919		.882
No	13.3 (6.7)		5.4 (6.0)		7.2 (6.0)		7.3 (6.6)		8.0 (6.6)	
Yes	16.3 (6.4)		5.4 (4.9)		7.1 (4.9)		7.1 (5.4)		8.3 (5.8)	
A4 fracture		.873		.878		.906		.817		.948
No	14.6 (6.9)		5.3 (5.9)		7.2 (5.8)		7.1 (6.4)		8.1 (6.6)	
Yes	14.3 (5.8)		5.6 (3.7)		7.0 (4.2)		7.6 (4.4)		8.3 (5.0)	

⁎ Imaging at discharge was obtained at 6.7 days (SD 8.6) postoperative.

† Imaging at 90 days was obtained at 96.5 days (SD 34.6) postoperative.

‡ Imaging after hardware removal and release of the temporarily stabilized motion segment was obtained at 167.5 days (SD 76.4).

§ Imaging at last follow-up was obtained at 1078.9 days (SD 876.9). Data is presented as mean (standard deviation) or count (percent).

We noticed a tendency for less postoperative progression of segmental kyphosis in the group with 2-staged, compared to single-staged bisegmental surgery (mean difference 3.1° after partial hardware removal, p=.064; 2.7° at last follow-up, p=.115). Female patients showed better postoperative preservation of segmental Cobb angle at all postoperative some points (mean difference 4.1° before discharge, p=.004; 3.4° at 90 days, p=.021; 3.5° after partial hardware removal, p=.049; 3.2° at last follow-up, p=.056). Patients with additional B- or C-type injury tended to have more segmental kyphosis before surgery (mean difference 3.0°, p=.086) but after surgery both groups showed similar values. There were no significant influences of additional B- or C-type injury, higher age or complete burst fracture (A4) type of the vertebral body on segmental kyphosis during follow-up (Table 3).

**Fig. 4:**
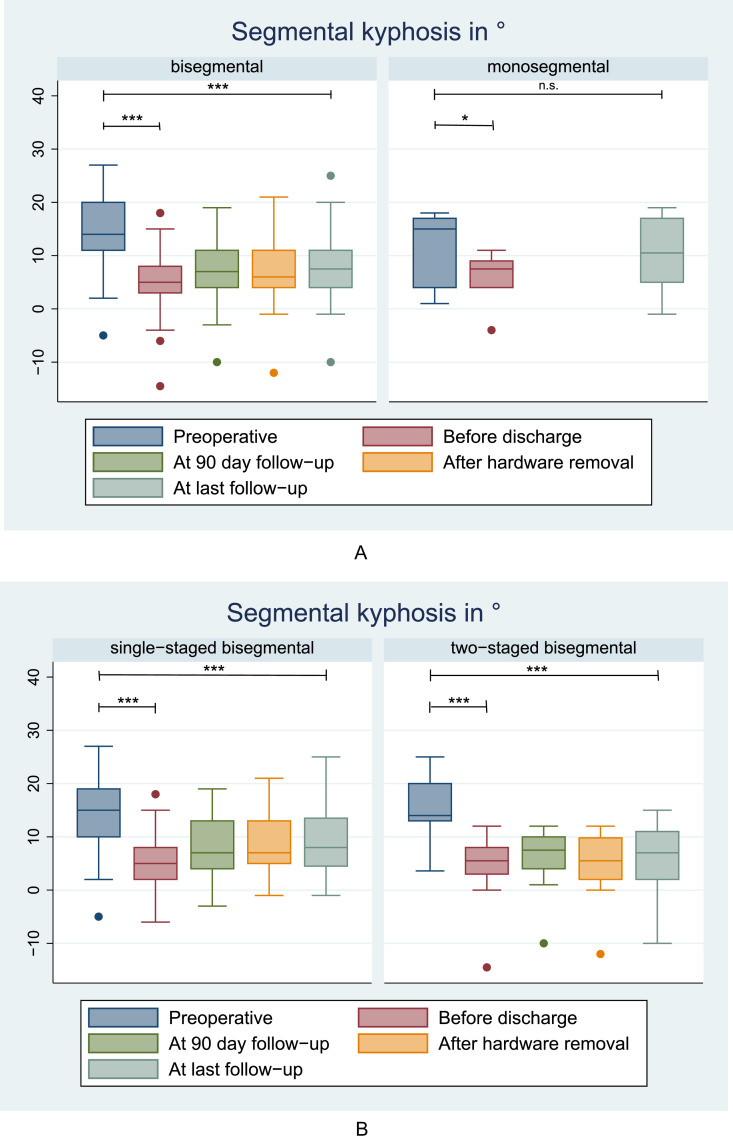
Box plots (median with 25th – 75th percentile, upper and lower percentiles [whiskers] and outliers [dots]) showing the segmental kyphosis angle (in degree; y-axis) of patients undergoing bisegmental instrumentation or primary monosegmental fusion (A) or of patients undergoing single- vs. 2-staged bisegmental instrumentation (B). Fig. 4A: Note the wider percentiles for the monosegmental fusion group for smaller group size (n=6 vs. n=55) but also less reproducible kyphosis reduction. Fig. 4B: Note the tighter percentiles in the 2-staged group, indicating a more reproducible kyphosis reduction. * = p<.05; *** = p<.001; n.s. = not significant.

The 6 patients receiving primary monosegmental fusion experienced good correction of kyphosis at time of discharge (mean difference 5.8°, p=.018), but progressive kyphosis and loss of Cobb angle correction during follow-up (mean difference preoperative to last follow-up 1.5°, p=.472; Fig. 4A). In n=55 patients receiving temporary bisegmental instrumentation/fusion, kyphosis correction was 9.5° at time of discharge (p<.001) and it persisted until last follow-up (mean difference 7.2°, p<.001; Fig. 4**A**).

Kyphosis reduction at time of discharge was highly significant in both, the subgroup with single-staged (mean difference 8.8°, p<.001) and the subgroup with 2-staged temporary bisegmental surgery (mean difference 10.6°, p<.001; Fig. 4B). Until 90 days, both groups lost some of the correction (single-staged: −1.8° [p<.001]; 2-staged: -1.7° [p=.001]). The single-staged group lost further 0.7° of sagittal Cobb angle after partial hardware removal on average (p<.001), while the 2-staged group gained 1.0° of segmental lordosis (p=.013)—likely due to the interbody cage placed during the same intervention. Until the last follow-up, further loss of sagittal Cobb angle was 0.7° in the single-staged (p<.001) and 1.1° in the 2-staged group (p=.003), respectively.

Regarding the different management types, we noticed the smallest change in sagittal Cobb angle / lowest progress in segmental kyphosis between discharge and last follow up in the 2-staged temporary bisegmental group (4.8° → 6.5°; mean difference −1.7°, p=.005). There was more progress in segmental kyphosis in the single-staged temporary bisegmental (5.9° → 9.1°; mean difference −3.2°, p<.001) and in the primary monosegmental group (5.8° → 10.2°; mean difference −4.3°, p=.052).

### Complications and outcomes

At 90 days, the clinical outcomes according to the MacNab criteria were favorable (excellent or good) in n=40 patients (65.6%). Postoperative complications at this time were noticed in 3 patients (4.9%), of which 2 required revision surgery (1 patient with wound infection and 1 with an aseptic wound necrosis; Table 4).

**Table 4:** Complications and clinical outcomes of n=61 patients who underwent lateral lumbar or thoracic interbody fusion (LLIF) for traumatic indications.

MacNab criteria at 90 days	
Excellent	10 (16.4%)
Good	30 (49.2%)
Fair	21 (34.4%)
Poor	- (0%)
Complications at 90 days	
No	58 (95.1%)
Yes*	3 (4.9%)
TDN grade of complications at 90 days	
Grade 1	1 (1.6%)
Grade 2	- (0%)
Grade 3	2 (3.3%)
n/a	58 (95.1%)
MacNab criteria at last follow-up†	
Excellent	40 (65.6%)
Good	18 (29.5%)
Fair	3 (4.9%)
Poor	- (0%)
Complications at last follow-up†	
No	50 (82.0%)
Yes‡	11 (18.0%)
TDN grade of complications at last follow-up†	
Grade 1	- (0%)
Grade 2	2 (3.3%)
Grade 3	9 (14.7%)
n/a	50 (82.0%)
Pseudarthrosis until last follow-up*	
No	60 (98.4%)
Yes	1 (1.6%)
**Total**	**n=61 (100%)**

⁎ Complications at 90 days were a wound hematoma (without need for revision surgery) and 2 wound complications (1 wound infection and 1 aseptic necrosis), which required revision surgery.

† The mean follow-up was 1078.9 days (SD 876.9).

‡ Complications at last follow-up were approach-related psoas weakness (n=1), low-grade infection of the posterior wound, requiring antibiotic treatment (n=2) or delayed screw/rod replacement (n=1), nonunion requiring posterior revision with iliac grafting (n=1), adjacent segment degeneration requiring surgical treatment (n=1). Five further patients wished complete hardware removal after confirmed fusion as they felt irritated by the screw heads. Data is presented as mean (standard deviation) or count (percent).

At time of last follow-up, favorable outcome was documented in n=58 patients (95.1%). Eleven patients (18%) experienced additional complications between 90 days and last follow-up, including 8 (13.1%) that required further spine surgery (Table 4). The reasons for revision surgery were patient requesting removal of the hardware (screws/rods; n=5), low-grade infection requiring delayed replacement of screws/rods (n=1), nonunion requiring posterior revision with iliac grafting (n=1), and adjacent segment degeneration requiring surgical treatment (n=1).

After a mean follow-up of 1078.9 days (SD 876.9) we identified pseudoarthrosis in 1 patient, corresponding to a fusion rate of 98.4%.

### Factors associated with outcome

At 90 days, male patients were less likely than female patients to achieve excellent or good outcome according to MacNab (OR 0.26, 95% CI 0.07–0.91, p=.035). Patients with a B-/C-type injury also tended to be less likely (OR 0.40, 95% CI 0.13–1.20, p=.100). Moreover, patients with an injury of the lumbar spine were more likely than patients with an injury of the thoracic spine to achieve excellent or good outcome (OR 8.57, 95% CI 1.03–71.35, p=.047). There was no significant association of outcome with either age or smoking status at 90 days.

At time of last follow-up, no significant association of any variable with the dichotomized clinical outcome according to MacNab could be established, as the rate of fair/poor outcomes at this time were too low.

## Discussion

In this retrospective, single-center series we report the short-, mid- and long-term results in terms of sagittal segmental angle, clinical outcomes, complications, and fusion rates in n=61 patients suffering from kyphosing fractures of the TL-junction and treated with “Trauma LLIF”. To the best of our knowledge, there are no publications in the scientific literature so far about the use of LLIF to improve the fusion rates and enable short-segment, monosegmental fusion in the setting of traumatic injuries of the thoracolumbar region. This series of consecutively treated patients at our center shows that the Trauma LLIF technique reliably promotes fusion and allows for a significant reduction of the fracture-induced segmental kyphosis. The segmental sagittal angles remain improved during long-term follow-up, however, with some loss of reduction over time.

### Why short-segment anterior-posterior fusion?

It is our philosophy to avoid long-segment instrumentation/fusion whenever possible, to maintain the mobility of noninjured motion segments and reduce the stress exerted on the implants/hardware on the one hand, and on the motion segments adjacent to the fusion on the other hand. Preserving as many motion segments as possible by limiting the number of fused segments constitutes a fundamental principle of spinal surgery as it minimizes alteration of spinal biomechanics and the risk of early degeneration of adjacent segments [6]. The patients in our cohort were the typical – young and predominantly male—trauma patients, who are usually working and taking care of family responsibilities [6]. Offering an effective type of treatment that interferes as little as possible with their previous obligations is desired. Owing to our long experience with lateral surgery for degenerative disc disease, deformity and corpectomies, applying the lateral approach to the TL-junction to promote fusion and reduce the risk for progressive kyphosis seemed logical. When applied with minimally-invasive (MIS)-techniques, the lateral approach to the disc requires a skin incision between 3-5cm and the rib usually does not need to be removed for mini-thoracotomies [19,26,27]. We consider the additional morbidity from the lateral approach in terms of disability, pain and blood loss/operation time as low and acceptable. Many patients feel discomfort from the thoracic drain, which can often be removed after 24h. On the contrary, being able to avoid long-segment fusion in this relatively young patient population is likely to be beneficial in the long run, even though this remains to be proven.

Our current results indicate high patient satisfaction with the received treatment, and high rates of favorable outcome at 90 days (65.6%) and even more so at 3 years postoperative (95.1%), according to MacNab criteria. The early complications (< 90 days after initial surgery) observed in our cohort all occurred in the posterior site (n=3 requiring revision surgery); no complication was related to the lateral approach or placement of the spacer. Complications after 90 days and until last follow-up included 1 patient with psoas weakness on the approach-side, but no other complications related to the LLIF and no LLIF spacer needed to be revised during follow-up (Table 4). Psoas weakness is a known complication after LLIF, in particular when approaching the L3/4 or L4/5 segments. It typically recovers over the course of a few weeks until 1 year [19,20,22,23,26,28,29]. At the TL-junction, the psoas is often very thin and weakness of the hip flexion a rarely observed complication.

The fusion and hence success rate in this series was 98.4%, which is similar (98.0%) [30] or superior (85.9%) [31] compared to other retrospective series of patients with thoracolumbar burst fractures treated with short-segment posterior only spinal fixation. In a series of n=18 patients with traumatic injury of the TL-junction undergoing monosegmental anterior column reconstruction with an expandable vertebral body replacement device, Lindtner et al. reported an average of 2.7° of segmental loss of correction and 5 patients (27.8%) with subsidence of the spacer during follow-up [6]. Studies on short-segment posterior fixation with use of a transforaminal lumbar interbody spacer for traumatic injuries of the TL-junction reported 2.9°–4.9° loss of segmental correction during follow-up and 16.7% of patients with hardware failure [32,33]. For the PLIF or TLIF approach to the disc, parts of the facet joints must be removed, which destabilizes the motion segment unnecessarily. Moreover, the PLIF or TLIF cages, but also expandable vertebral body replacement device, are often placed in the vulnerable region of the injured endplate, which may lead to progressive cage subsidence during follow-up [6]. The LLIF technique offers the advantages that no unnecessary destabilization must be performed, and the large spacer spanning the apophysis of the vertebral body prevents from subsidence and associated pseudarthrosis [20,26]. We anticipate future studies to provide better evidence regarding the comparative long-term outcomes of posterior-anterior monosegmental versus posterior short-segment instrumentation/fusion and of different posterior-anterior fusion types (LLIF vs. TLIF/PLIF).

### Which “trauma LLIF” technique is the best?

During the past twelve years, while applying the Trauma LLIF technique in our center, we have gained the impression that the most reliable results are obtained by a 2-staged, temporary bisegmental procedure (Fig. 3). The present results support our notion, even though in direct comparison both techniques entailing temporary bisegmental instrumentation (single- and 2-staged) showed effective prevention of kyphosis (Table 3 and Fig. 4).

As evident from Fig. 4B, segmental kyphosis at last follow-up was significantly reduced for both temporary bisegmental techniques, compared to the preoperative baseline measurement. It is visible in the graphs that the median segmental kyphosis value increases steadily over time in the single-staged group, whereas in the 2-staged group the kyphosis angle decreases between 90 days postoperative (after the posterior & before the lateral procedure) and after partial hardware removal, which was always combined with the LLIF procedure in the same anesthesia (Figs. 1 and 3). The introduction of the LLIF implant hence results in some distraction of the anterior column, which in turn decreases the segmental kyphosis. At this time, we noticed an almost significant difference in segmental kyphosis between both bisegmental groups (p=.064; Table 3). The mean difference in segmental kyphosis between the single-staged and 2-staged temporary bisegmental group was <1° at discharge, 1.3° at 90 days, increasing to 3.1° after partial hardware removal/delayed LLIF and 2.6° at time of last follow-up; all small values that may be clinically irrelevant in young patients with flexible spines and healthy compensatory mechanisms. In summary, both temporary bisegmental techniques seem to work (Fig. 3)—with LLIF conducted immediately during the index hospitalization or in a delayed fashion at time of partial hardware removal (3–5 months after the index surgery).

We noticed more heterogenous results in the subgroup of patients that were treated by primary monosegmental posterior-anterior fusion without temporary instrumentation of the next lower motion segment (Fig. 4A). Here, the benefit is that after the index procedure, patients require no further delayed surgical procedure for hardware removal or placement of the spacer (Fig. 3). This type of management is appealing in young adults that wish to return to work or their other obligations without disruption by additional hospitalizations. We have made the experience, however, that even though the injured segment solidly fused during follow-up, the initial correction of segmental kyphosis was often lost. Therefore, this type of treatment may work in patients with little fracture comminution and excellent bone quality, but our results obtained in patients receiving temporary instrumentation to the next lower motion segment seem better. Even though in Table 3, no difference in segmental kyphosis was evident between both techniques during follow-up, the small sample size in the primary monosegmental group (n=6) results in insufficient power to confirm our observation with statistical significance.

### The influence of further factors on segmental kyphosis

We found no strong impact of higher age on segmental kyphosis (Table 3). However, as our total cohort was fairly young with a median age of 36 years, it is not surprising that patients in the “elderly” group fared equally well. Sensitivity analyses stratifying the group by an age cutoff of 50 years found that patients aged 50 years or higher (n=17) had slightly worse segmental kyphosis at all time points during follow-up (MD −1.6° at discharge, MD −1.1° at 90 days, MD −1.6° after partial hardware removal and −2.5° at last follow-up, all p>.05). The data therefore suggest that the Trauma LLIF technique works similarly well in patients at younger and more advanced age groups.

Both male and female patients showed similar segmental kyphosis values before surgery, but the reduction of kyphosis was more effective in female patients at time of discharge (MD −4.1°, p=.004) and remained stable at 90 days (MD −3.5°, p=.024), after partial hardware removal (MD −3.4°, p=.049) and by trend at time of last follow-up (MD −3.2°, p=.056). The reasons for this observation are not fully clear to us, as we could not identify male sex as risk factor for post-traumatic kyphosis in the literature [34]. The distribution of the injured spinal levels was similar; hence the observed difference is not explained by confounding spinal geometry. However, female patients in our cohort were about 6.7 years younger than male patients (34.8 vs. 41.6 years, p=.052), which may have contributed to this effect. We desisted from a more in-depth analysis, as the scope of this article was different.

We observed no unfavorable result in terms of segmental kyphosis correction in patients with more unstable B- or C-type injuries, compared to A-type injuries only (Table 3). Comparative sagittal Cobb angles at all time points during follow-up indicate that the Trauma LLIF technique is suitable for simple A-type, but also for more severe injury types. There were n=24 patients with B-type injuries included in the analysis, but only 2 patients with C-type injuries. Hence, some uncertainty remains for this latter category and more data in future studies is required to confirm the applicability of Trauma LLIF in patients with C-type injuries of the TL-junction.

Regarding complete burst fractures, some words of caution are also required. Our analysis reveals that the segmental sagittal Cobb angle was similar for A4 (n=11), compared to A1-A3 fractures (n=50; Table 3). As mentioned in the methods section, we did consider the Trauma LLIF technique in selected patients with superior burst-split fracture (A3.2.1, according to Magerl) [3]. However, a substantial portion of patients with A4 fractures according to the AO Spine classification^4^ that appeared with high comminution of the vertebral body [35], including also a more pronounced fissure containing ruptured disc material, were treated by bisegmental fusion and lateral corpectomy [6]. The fact that our included patients with A4 fractures showed no worse results (Table 3) likely confirms that we may have selected those patients adequately, but we advise against treating A4 fractures with severe comminution of the vertebral body with the Trauma LLIF technique.

### Strengths and weaknesses

This paper reports on a novel technique to reliably promote monosegmental fusion and reduce kyphotic deformity after traumatic injury in the TL-junction. The reasonably large sample size, paired with a low missing-data burden and long follow-up allows for solid conclusions pertaining to the main outcome variables. Moreover, besides the radiological measures, we included an assessment of complications and clinical outcomes at 90 days and last follow-up.

The lack of PROMs is a weakness, but PROMs were only introduced in our center in January of 2022, and this information was missing for most patients included in this retrospective study. Information on return-to-work would have been interesting, especially to compare single-staged with 2-staged procedures, but also this information could not be obtained in all patients in this retrospective study. Also, even though we noticed statistical trends in subgroup analyses, the sample sizes may have not been sufficient to determine the ideal surgical strategy with sufficient statistical power. The time of the last follow-up differed between patients, and while it was multiple years in the initially treated patients it was shorter in those more recently operated (average follow-up about 3 years). Lastly, our analysis does not contain a control group of patients treated with either posterior only short- or long-segment fixation/fusion or any other form of short-segment posterior-anterior fusion (e.g., TLIF, PLIF), hence a direct comparison to an alternative treatment is not possible.

### Implications for practice

We suggest that surgeons who are trained in lateral surgery to the thoracolumbar spine consider short-segment posterior fixation/fusion with Trauma LLIF as surgical treatment option for traumatic injuries of the TL-junction (Th10/11-L2/3). Suitable injury types include A3 fractures in general, as well as selected A4 fractures with low fracture comminution [35], where a single simple split fracture line of the fractured vertebral body (without disc material ruptured into the vertebral body) extends down to the lower disc level (A3.2.1, according to Magerl) [3,6]. Our data suggest that B-type injuries can be treated similarly well as A-type injuries; whether short-segment posterior-anterior fixation/fusion by Trauma LLIF is sufficient for all C-type injuries is currently not sufficiently clear. Based on the experiences made over the past 12 years with this technique, we advise to be cautious with primary monosegmental fusion, as we observed some loss of segmental correction over the follow-up time. Both techniques that entail temporary bisegmental instrumentation – single-staged and 2-staged LLIF (Fig. 3) – lead to significant and lasting correction of posttraumatic segmental kyphosis, high rates of fusion and favorable outcome.

## Conclusions

“Trauma LLIF” should be considered as possibility for short-segment anterior-posterior fusion for injuries of the thoracolumbar junction. Of several different management options tried over the past years, we observed most reproducible and long-lasting kyphosis reduction with a temporary bisegmental, 2-staged procedure (posterior instrumentation fusion with delayed LLIF and hardware removal to release the noninjured caudal motion segment). More data, especially on clinical outcomes, treatment-related complications and return-to-work are needed to compare Trauma LLIF against alternative treatment options for injuries of the thoracolumbar junction.

## Declaration of generative AI and AI-assisted technologies in the writing process

No AI or AI-assisted technologies were used for the writing process of this article.

## Funding statement

No funding was received for this research.

## Declarations of competing interest

The authors declare that they have no known competing financial interests or personal relationships that could have appeared to influence the work reported in this paper.
